# The Female Heart: Sex Differences in the Dynamics of ECG in Response to Stress

**DOI:** 10.3389/fphys.2018.01616

**Published:** 2018-11-28

**Authors:** Tricia Adjei, Jingwen Xue, Danilo P. Mandic

**Affiliations:** Communications and Signal Processing, Department of Electrical and Electronic Engineering, Imperial College London, London, United Kingdom

**Keywords:** male stress, female stress, cognitive stress, ECG, HRV, entropy and complexity, LF and HF in HRV

## Abstract

Sex differences in the study of the human physiological response to mental stress are often erroneously ignored. To this end, we set out to show that our understanding of the stress response is fundamentally altered once sex differences are taken into account. This is achieved by comparing the heart rate variability (HRV) signals acquired during mental maths tests from ten females and ten males of similar maths ability; all females were in the follicular phase of their menstrual cycle. For rigor, the HRV signals from this pilot study were analyzed using temporal, spectral and nonlinear signal processing techniques, which all revealed significant statistical differences between the sexes, with the stress-induced increases in the heart rates from the males being significantly larger than those from the females (*p*-value = 4.4 × 10^−4^). In addition, mental stress produced an overall increase in the power of the low frequency component of HRV in the males, but caused an overall decrease in the females. The stress-induced changes in the power of the high frequency component were even more profound; it greatly decreased in the males, but increased in the females. We also show that mental stress was followed by the expected decrease in sample entropy, a nonlinear measure of signal regularity, computed from the males' HRV signals, while overall, stress manifested in an increase in the sample entropy computed from the females' HRV signals. This finding is significant, since mental stress is commonly understood to be manifested in the decreased entropy of HRV signals. The significant difference (*p*-value = 2.1 × 10^−9^) between the changes in the entropies from the males and females highlights the pitfalls in ignoring sex in the formation of a physiological hypothesis. Furthermore, it has been argued that estrogen attenuates the effect of catecholamine stress hormones; the findings from this investigation suggest for the first time that the conventionally cited cardiac changes, attributed to the fight-or-flight stress response, are not universally applicable to females. Instead, this pilot study provides an alternative interpretation of cardiac responses to stress in females, which indicates a closer alignment to the evolutionary tend-and-befriend response.

## 1. Introduction

The effects of stress on heart rate (HR) and heart rate variability (HRV) are considered to be well defined and long established. General physiological stress is understood to cause an increase in HR, consequently decreasing HRV (Houtveen et al., 2002), whilst physical stress has been widely reported to cause an increase in the power of the low frequency (LF) component of HRV signals, and a decrease in the power of the high frequency component (Montano et al., 1994). It is therefore commonly conjectured, though not without controversy, that the LF component of HRV (0.04–0.15 Hz) reflects the activity of the sympathetic nervous system (SNS), while the HF component (0.15–0.4 Hz) reflects the activity of the parasympathetic nervous system (PNS). The controversy surrounding this conjecture was highlighted in several studies where the effects of mental stress on the LF and HF components of HRV were found to be neither consistent, nor correlate with the trends seen in physical stress (Berntson et al., 1994). However, despite the uncertainty over the physiological interpretations of the LF and HF components, and whether or not they, respectively, represent the activities of the SNS and PNS (Berntson et al., 1994; Eckberg, 1997; Berntson and Cacioppo, 2004; Billman, 2013; von Rosenberg et al., 2017), it comes as a surprise that until recently, the theories regarding the relationships between stress, HR and HRV were developed without accounting for, or appreciating sex differences. To this end, this work aims to quantify and demystify the effects of sex differences on the dynamics of ECG, in a proof-of-concept study based on ECG recordings from participants who have been subjected to a mental stressor.

The commonly adopted theory of the stress response stems from a seminal book by Walter Cannon, in which it was hypothesized that, irrespective of sex, the evolutionary purpose of the human stress response is to prepare the body for *fight* or *flight* when faced with a threat (Cannon, 1915). It is generally accepted that reaching either the fight or flight stage requires the energization of the body through stress hormones, for example, by increasing HR, arousal and the respiratory rate. However, long-term exposure to these hormones is known to lead to pathologies, such as heart disease, insomnia and hyperventilation (NHS Derbyshire Health Psychology Service, 2012). Chronic stress is also linked to depression; it is known that chronic stress causes structural degeneration in the pre-frontal cortex, which is a major risk factor for depression (Mah et al., 2016). It would therefore be expected that the correlations between many stress induced pathologies, such as heart disease and depression, would be high; instead, the statistics indicate no such correlation (Åhs et al., 2009).

Whilst females have the highest incidence of depression, the highest incidence of cardiac pathologies is seen in males. For example, in 2014, 4.3% of UK females had experienced depressive episodes, compared to 3.2% of males (NHS Digital, 2016). In contrast, in the year 2013/14, the number of UK males admitted to hospital with heart related diseases was 30.3% higher than the corresponding number of females (British Heart Foundation, 2015). Such stark statistical differences have led to further investigation into the causes of this disparity. An insightful overview of sex differences in the response to stress by Verma et al. (2011) reports many hormonal, neuroanatomical and cognitive differences between the sexes; it concludes that males and females exhibit distinct psychological and biological differences in their responses to stress. Similarly, Ramaekers et al. (1998) assessed sex differences in cardiovascular dynamics in response to stress, by analysing 24-h-long HRV signals, recorded from 135 females and 141 males aged between 18 and 71 years. They reported that the absolute powers of the LF components of HRV from the males, regardless of age, were significantly larger than those from the females aged under 40, but were not significantly larger than those from the females aged over 40 (Ramaekers et al., 1998). This age-dependence has indicated that the possible sex difference in cardiovascular dynamics is due to the effects of the menstrual cycle, and in particular, estrogen (Ramaekers et al., 1998). It is now hypothesized that estrogen enhances parasympathetic control of the heart (Dart et al., 2002), which in turn means that premenopausal females will experience enhanced parasympathetic control compared to males and postmenopausal females. This gives a wide scope for the study of the effect of estrogen on cardiovascular dynamics, which promises to dramatically alter the understanding of the stress response, a subject of this work.

At present, the effect of hormones on the cardiovascular response to stress is considered to be related to the activation of the sympathetic nervous system (SNS), which mediates the release of catecholamines and glucocorticoids (Lundeberg, 2005). Many researchers have posited that once a stressor diminishes, the parasympathetic nervous system (PNS) takes dominance over the SNS (Figueroa-Fankhanel, 2014); the PNS is known to restore vital functions to their rest state (Thayer et al., 2009). However, the theory that the SNS and PNS have an antagonistic or reciprocal relationship has also become controversial (Billman, 2013). An often cited review by Eckberg (1997) assesses SNS and PNS dynamics, and reports many parallel activations of the SNS and PNS, and that there is no definitive physiological evidence to suggest that the SNS and PNS must behave reciprocally. Eckberg (1997) describes the tendency to assign a reciprocal relationship to the SNS and PNS as philosophical, as opposed to physiological. Another study often cited to support nonreciprocal SNS and PNS dynamics is Berntson et al. (1994), who assessed the SNS and PNS responses in 10 participants subjected to mental stress, and reported slightly positive correlations between the activities of the SNS and PNS. They concluded that certain stressors may elicit autonomic responses which are specific to individuals, whilst others elicit common SNS and PNS responses. Yet, despite the popularity of the paper, it is often overlooked that all 10 of the subjects recruited by Berntson et al. (1994) were young females. In light of the work by Dart et al. (2002), a study cohort made up entirely of young females, without an account of their menstrual cycle phase, could explain why the results reported by Berntson et al. (1994) differed from similar studies. In summary, conflicting findings regarding the cardiovascular responses to mental stress point to a lack of clarity in the understanding of the stress response; this motivated us to ask whether this lack of clarity was simply due to conflating the male and female responses to stress?

To provide a conclusive answer to this question, we set out to assess the cardiovascular dynamics of young males and young females during a mental stress task; this was achieved in a rigorous, quantitative, and reproducible way, by applying state-of-the-art signal processing techniques to HRV signals. We make no attempt to correlate the LF and HF components of HRV to the SNS and PNS, but to identify differences in the temporal, spectral and nonlinear characteristics of the recorded signals. The changes in heart rate will be used to characterize signals temporally, whilst spectral characterization is achieved through the computation of the powers of the LF and HF components of HRV, and nonlinear characterization is accomplished using the nonparametric sample entropy (SE) method. Our results show conclusively that males and females in the follicular phase of their menstrual cycle exhibit significantly different cardiovascular responses to mental stress.

## 2. Materials and methods

### 2.1. Subjects

Our study cohort consisted of 10 males and 10 females, with respective mean ages of 28.6 (standard deviation: 5.6, range: 23–38) and 23.3 (standard deviation: 1.3, range: 21–26). The size of the dataset used is validated by a statistical study (Ristic-Djurovic et al., 2018), which reported that nine is the minimum recommended number of subjects to draw statistically significant conclusions from a biomedical study.

The calendar method was used to confirm that all female subjects were in the follicular phase of their menstrual cycle; the follicular phase precedes ovulation, and is characterized by increasing estrogen levels (Dart et al., 2002).

The experimental procedure was explained to the subjects, both verbally and in writing, and all subjects gave their full consent to take part in the study. Ethics approval was granted by the Joint Research Office at Imperial College London (reference IRREC_12_1_1).

### 2.2. Experimental procedure

The electrocardiogram (ECG) was recorded from each subject as they sat at rest for 15 min, and during a 15-min mental maths test in pairs (subjects competed against sex-matched opponents with similar mathematical abilities and proficiency in the English language). There was a 1-min interval between the rest and test periods, and to induce further mental stress, the subjects were told that their performance in the maths test would be recorded.

### 2.3. Data acquisition

The subjects' ECGs were recorded by a custom-made data logger, called the iAmp (Kanna et al., 2018), using adhesive surface electrodes placed in the Lead I ECG configuration, and at a sampling frequency of 1,000 Hz.

All signal processing was performed in the Matlab programming environment. The ECG signals from the subjects were segmented into epochs, corresponding to the rest and mental maths test, before HRV was derived. The R-waves in the ECGs were extracted and interpolated at 4 Hz to derive HRV, using the robust algorithm introduced in Chanwimalueang et al. (2015). Temporal, spectral and nonlinear signal processing techniques were applied to the epochs of HRV to extract the four cardiac metrics described below.

#### 2.3.1. Temporal analyses

The HR from the HRV signals was computed from a 1-min sliding window, with a one-second increment, where *x*_*i*_ denotes an HRV data point in the windowed signal, and *n* designates the number of data points in the window, to yield

(1) $$\begin{array}{l} HR=\frac{\sum_{i\;=\;1}^{n}\left(\frac{60}{x_{i}}\right)}{n} \end{array}$$

#### 2.3.2. Spectral analyses

The powers of the LF and HF bands in HRV were computed as the powers of the 0.04–0.15 Hz and 0.15–0.4 Hz bands, respectively, and were normalized by the power of the 0.04–0.5 Hz band, *N*_*p*_, to give

(2) $$\begin{array}{lll} Normalized\;LF & = & \frac{LF_{p}}{N_{p}} \end{array}$$

(3) $$\begin{array}{lll} Normalized\;HF & = & \frac{HF_{p}}{N_{p}} \end{array}$$

where the respective powers of the LF and HF bands are denoted by *LF*_*p*_ and *HF*_*p*_.

The analysis was performed within a 5-min sliding window, with a one-second increment. This window duration is the minimum recommended length to fully capture spectral cardiac dynamics (Malik et al., 1996).

This new *N*_*p*_ power band of interest was used in Equations (2) and (3) for the normalization, instead of the total power for the following reasons: (i) it has long been known that the frequencies below 0.04 Hz have no clear physiological interpretation (Malik et al., 1996), and (ii) heart beats are produced at a maximal rate of 1 Hz, which means the effective sampling frequency of HRV is also 1 Hz; the Nyquist theorem therefore suggests all useful information in HRV is contained below 0.5 Hz (Kuusela, 2004).

The mean normalized powers of the LF and HF bands were computed for both the rest and mental maths epochs, for each subject. The mean normalized powers of the LF and HF bands will be simply referred to as LF and HF, respectively.

#### 2.3.3. Nonlinear analyses

Numerous studies have investigated the effect of stress on the structural complexity within HRV signals. Central to the analysis of signal complexity is the complexity loss theory as introduced by Goldberger et al. (2002), which posits that any perturbations within a physiological system, such as those caused by stress, constrain the system. Goldberger et al. (2002) hypothesize that the signals recorded from a constrained physiological system will exhibit reduced structural complexity. Signal complexity is interpreted through the regularity of a signal and is measured using entropy algorithms; equating structural complexity to signal regularity justifies the use of entropy to analyse signal complexity, as entropy algorithms are widely used measures of regularity. However, a truly complex system is neither completely regular nor irregular (Tononi et al., 1998), which possibly renders entropy an inadequate measure of complexity (Goldberger et al., 2002).

Nevertheless, many studies which use entropy to assess the effects of stress on signal complexity have concluded that stress reduces the complexity within cardiac signals, supporting the complexity loss theory. Vuksanovic and Gal (2007), Williamon et al. (2013), and Chanwimalueang et al. (2016) employed the sample entropy method to analyse the effect of mental stress on the structural complexity in HRV signals, and found that mental stress led to a decrease in entropy. Bornas et al. (2006) employed sample entropy to analyse ECG signals from flight phobics, and found that sitting in a flight simulator resulted in a reduction in the entropy of their ECG. Given the choice of the sample entropy method over other entropy measures in these relevant studies, we here apply sample entropy to assess sex differences in the effects of stress on the structural dynamics of HRV signals.

The sample entropy algorithm was introduced by Richman and Moorman (2000), and is the negative natural logarithm of the likelihood that two similar segments of data will remain similar, within a given tolerance, if the lengths of the segments are increased by one data point; see Step 1 to Step 7 in Algorithm 1 (Costa et al., 2002, 2005; Song et al., 2012). The sample entropy analyses in this study were undertaken in a 5-min sliding window, with a one-second increment; mean SE values for every subject for the two experiment epochs were computed.

**Table:** 

**Algorithm 1**: Sample Entropy	
1	A normalized signal, *x*, of length *N* is split using an embedding dimension, *m*, to create (*N*−*m*+1) segments. Each segment, *X*_*m*_, is of length *m*. In this study, *m* was defined as *m* = 2.
2	A tolerance level of *r* = 0.15 × *std*, where *std* is the standard deviation of the segment, is defined.
3	The maximum difference, *d*_*max*_, between the elements of two consecutive segments, *X*_*m*_(*i*) and *X*_*m*_(*j*), is computed as (4) $$\begin{array}{l} d_{max}=\underset{k\;=\;0,...,m-1}{max}(\left|x(i+k)-x(j+k)\right|) \end{array}$$
4	For each *X*_*m*_(*i*), the event *d*_*max*_<*r* is defined as a match, and a count of such matches is denoted by *A*_*i*_. The probability of matches, $A_{i}^{m}\left(r\right)$, for *X*_*m*_(*i*) is calculated as (5) $$\begin{array}{l} A_{i}^{m}=\frac{A_{i}}{N-m-1} \end{array}$$ Note: The denominator, *N*−*m*−1, ensures that when *m* is increased to *m*+1, the segment *X*_*m*+1_(*i*) is accounted for.
5	Then the sum of the probability of matches for all segments, Φ, is defined as (6) $$\begin{array}{l} \Phi^{m}(r)=\frac{\sum_{i\;=\;1}^{N-m}A_{i}^{m}(r)}{N-m} \end{array}$$
6	The embedding dimension *m* is increased to (*m*+1), and Step 1 to Step 5 are repeated; the sum of the probability of matches for all segments when *m* = *m*+1 are defined as Ψ, and the sample entropy, *SE*(*m, r*), is computed as (7) $$\begin{array}{l} SE(m,r)=ln(\frac{\Phi }{\Psi }) \end{array}$$

#### 2.3.4. Statistical analyses

The percentage changes in the above described cardiac metrics, from the rest to maths epochs, were used to compare the male and female cardiovascular reactions to mental stress. The use of percentage changes enables inter-subject statistical comparisons, whilst the choice of the statistical test employed was dependent on the distribution of percentage changes. For example, Student's *t*-test was used to compare the results which followed a normal distribution, and the Wilcoxon rank-sum was used to compare the results which did not follow a normal distribution. A significance level of 0.01 was assumed in all tests.

## 3. Results

**Figure 1:**
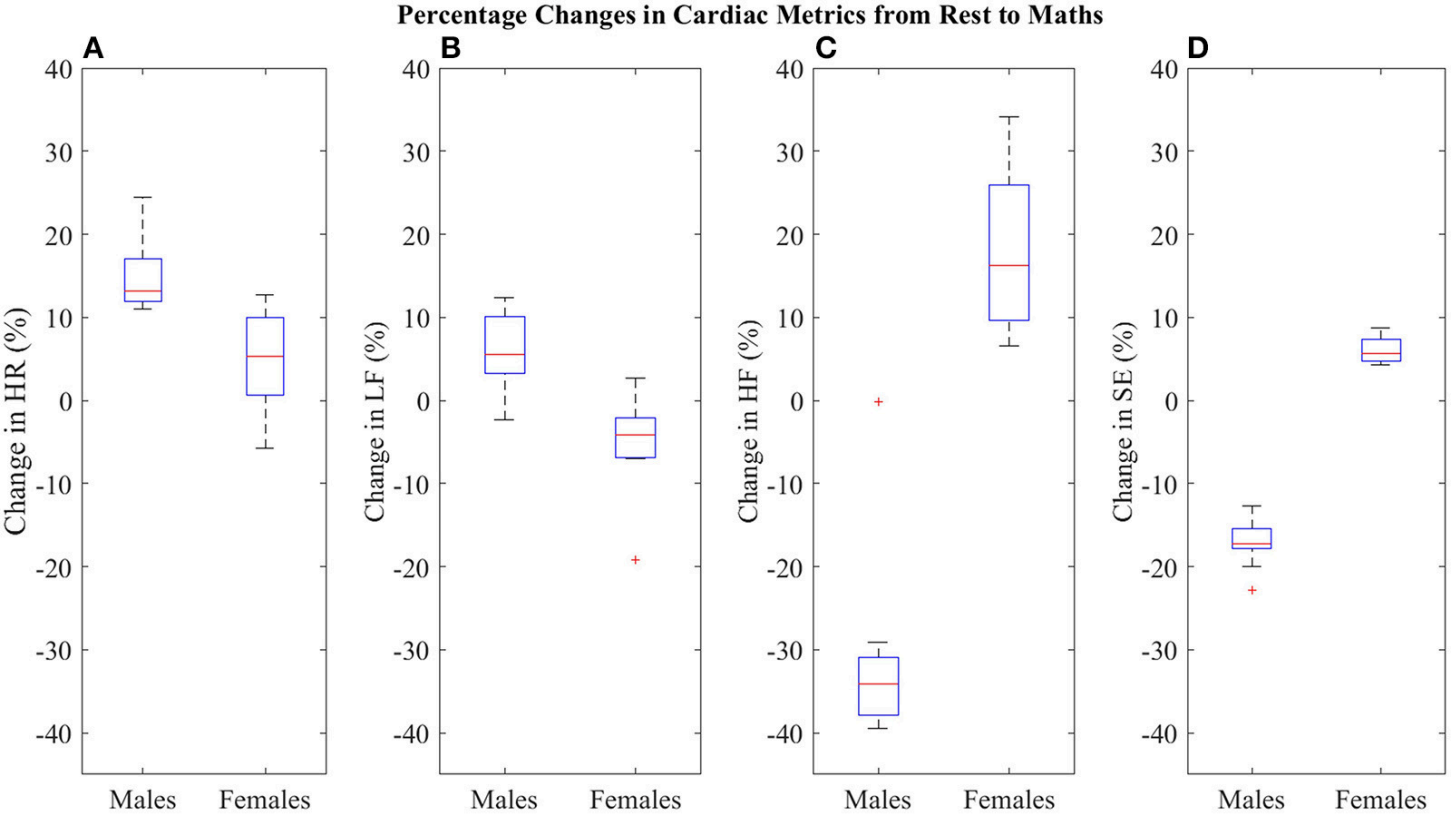
Temporal, spectral and nonlinear analyses of HRV. **(A)** Distribution of percentage changes in HR from the rest to maths epochs. **(B)** Distribution of percentage changes in LF from the rest to maths epochs. **(C)** Distribution of percentage changes in HF from the rest to maths epochs. **(D)** Distribution of percentage changes in SE from the rest to maths epochs.

Figure 1A shows that mental stress induced a significantly greater increase in HR in the males, compared to the females (Wilcoxon rank-sum: *p*-value = 4.4 × 10^−4^). The median increase in HR in the males was 13%, with a range of +11% to +25%; in contrast, the median percentage change in the females was an increase of only 5%. The changes in HR in the females were also less consistent, with a range of −7 to +13%. The median changes in the cardiac metrics and the *p*-values indicating the significance of the differences between the male and female responses are shown in Table 1.

**Table 1:** The percentage changes in the cardiac metrics from the rest epochs to the maths epochs.

**Median percentage changes in the cardiac metrics**			
**Metric**	**Males (%)**	**Females (%)**	*p***-value**
HR	+13	+5	4.4 × 10^−4^
LF	+5	−4	4.6 × 10^−4^
HF	−34	+16	1.8 × 10^−4^
SE	−17	+6	2.1 × 10^−9^

Figures 1B,C illustrate the sex differences in the cardiac responses to mental stress. The results from the spectral analyses show a non-reciprocal relationship between LF and HF; the median changes in LF and HF in the males were a respective 5% increase and a 34% decrease, whilst the corresponding changes in the females were a 4% decrease in LF and a 16% increase in HF.

The percentage changes in LF from the males were significantly different to those from the females (*t*-test: *p*-value = 4.6 × 10^−4^), and were more varied, with a range of −2 to +12%; those from the females ranged from −7 to +3%. In addition, the percentage changes in HF were far more distinguishing (Wilcoxon rank-sum: *p*-value = 1.8 × 10^−4^). The changes in HF in the males were more concentrated, with a range of −40 to −30%, whereas the changes in the females ranged from +7 to +34%.

Figure 1D shows the findings from the nonlinear analysis, which revealed the most significant difference between the male and female cardiac responses to mental stress (*t*-test: *p*-value = 2.1 × 10^−9^). The median percentage change in SE in the males was a 17% decrease, with a range of −20 to −13%. The median percentage change in the females was a 6% increase in SE, with a range of a +4 to +9%.

## 4. Discussion

The changes in HR shown in Figure 1A provide the first conclusive evidence that the expected increase in HR in response to stress is not universal; while every male experienced an increase in HR of at least 11%, only half of the females experienced increases of at least 5%. *The results from this study therefore confirm that the effect of stress on cardiac dynamics can differ substantially between males and females; this calls for a re-evaluation of our understanding of how stressful events affect cardiac dynamics in females in the follicular phase of their menstrual cycle*. A similar sex difference has previously been reported in Tousignant-Laflamme et al. (2005) in relation to the effects of pain (a form of physiological stress) on HR. It was found that whilst the correlation between pain intensity and HR was positive in males, no such correlation existed in the females (Tousignant-Laflamme et al., 2005). However, Tousignant-Laflamme et al. (2005) did not ascertain the menstrual cycle phase of their subjects, and hence, were not able to make inferences regarding the effect of sex hormones on the cardiac responses to pain. In addition, a meta-analysis into sex differences in HRV was conducted by Koenig and Thayer (2016), and it was concluded that HRV in females contains more power in the high frequency component (the effect of stress on HRV was not included in the investigation).

The effect of estrogen on the action of catecholamines has been widely investigated in mammals. In a review of the effect of estrogen on the stress response, Ueyama et al. (2008) reported that HR increases which were induced by stress in ovariectomised rats were greater than those experienced by ovariectomised rats who were supplemented with estrogen. Ueyama et al. (2008) hypothesized that their results were due to estrogen reducing the sympathoadrenal outflow of stress hormones from the central nervous system. Ueyama et al. (2008) also reported that estrogen reduced the reactivity of the heart to catecholamines, protecting the heart from the effects of stress. If applied to humans, these findings from rats would explain why females in this present study experienced smaller increases in HR when stressed.

Furthermore, the results from the spectral analyses in this study also reveal sex differences. Not only did the males and females exhibit contrasting changes in LF and HF, but the changes in HF were considerably larger than the corresponding changes in LF (see Table 1). *The overall stress-induced decrease in LF and the increase in HF in the females is a finding that contradicts the conventional understanding of the relationships between stress and the low frequency and high frequency components of HRV*. As already mentioned, studies such as Berntson et al. (1994) have previously indicated a lack of consistency in the LF and HF responses to stress after comparing their findings from all-female study cohorts to findings from studies with male cohorts. It is notable that these conclusions were drawn at a time when there was little awareness of sex differences in cardiac dynamics. Therefore, irrespective of the physiological interpretations of LF and HF, the opposing LF and HF trends in males and females, discovered in our pilot study, suggest that the inconsistencies reported by Berntson et al. (1994) were probably due to sex differences, and not a redundancy in LF and HF as stress metrics.

It can also not be ignored that the controversy over the physiological interpretation of LF and HF remains largely unresolved. In summary, LF has been speculated to: (i) represent the modulation of both the SNS and PNS (Malik et al., 1996), (ii) be influenced by the frequencies of slow breathing (Brown et al., 1993), and (iii) be influenced by the frequency of muscle contractions in blood vessels (Kenwright et al., 2009). Similarly, HF has also been suggested to be influenced by typical respiratory frequencies (Kenwright et al., 2009). In conclusion, the physiological interpretation of LF and HF cannot be verified without conducting an extensive endocrinological study into the relationship between mental stress, LF, and HF, in which respiration is controlled (Stacey et al., 2018).

Also, the spectral results reported here support the LF and HF normalization method employed in this study. The more common normalization methods are shown in Equations (8) and (9) below.

(8) $$\begin{array}{l} Normalized\;LF=\frac{LF_{p}}{LF_{p}+HF_{p}} \end{array}$$

(9) $$\begin{array}{l} Normalized\;HF=\frac{HF_{p}}{LF_{p}+HF_{p}} \end{array}$$

Observe that both of these normalizations would contain the same information, as in this way *LF*_*p*_ = 1−*HF*_*p*_ (Burr, 2007). In contrast, Figures 1B,C establish that the normalized LF and HF metrics proposed in this study contain different information, whereby relatively small changes in LF can be seen alongside large changes in HF. It is evident that the analysis of HRV via LF and HF provides an additional degree of freedom, in comparison to the single degree of freedom offered by HR analysis; without the two degrees of freedom, the sex-specific trends in LF and HF would not have been seen.

The results from the nonlinear analyses in this study also shed new light on the sex differences in the dynamics of ECG. Bornas et al. (2006), Vuksanovic and Gal (2007), Williamon et al. (2013), and Chanwimalueang et al. (2016) have all reported that mental stress causes decreases in the entropy of cardiac signals, however, our results in Figure 1D demonstrate that stress caused increases in the entropies computed from the female subjects. These results contradict the complexity loss theory from Goldberger et al. (2002), possibly supporting their view that entropies are not true measures of physiological complexity. Nevertheless, the decreased regularity of the HRV from the female subjects confirm that they experienced a stress response which differed from that of the males.

**Figure 2:**
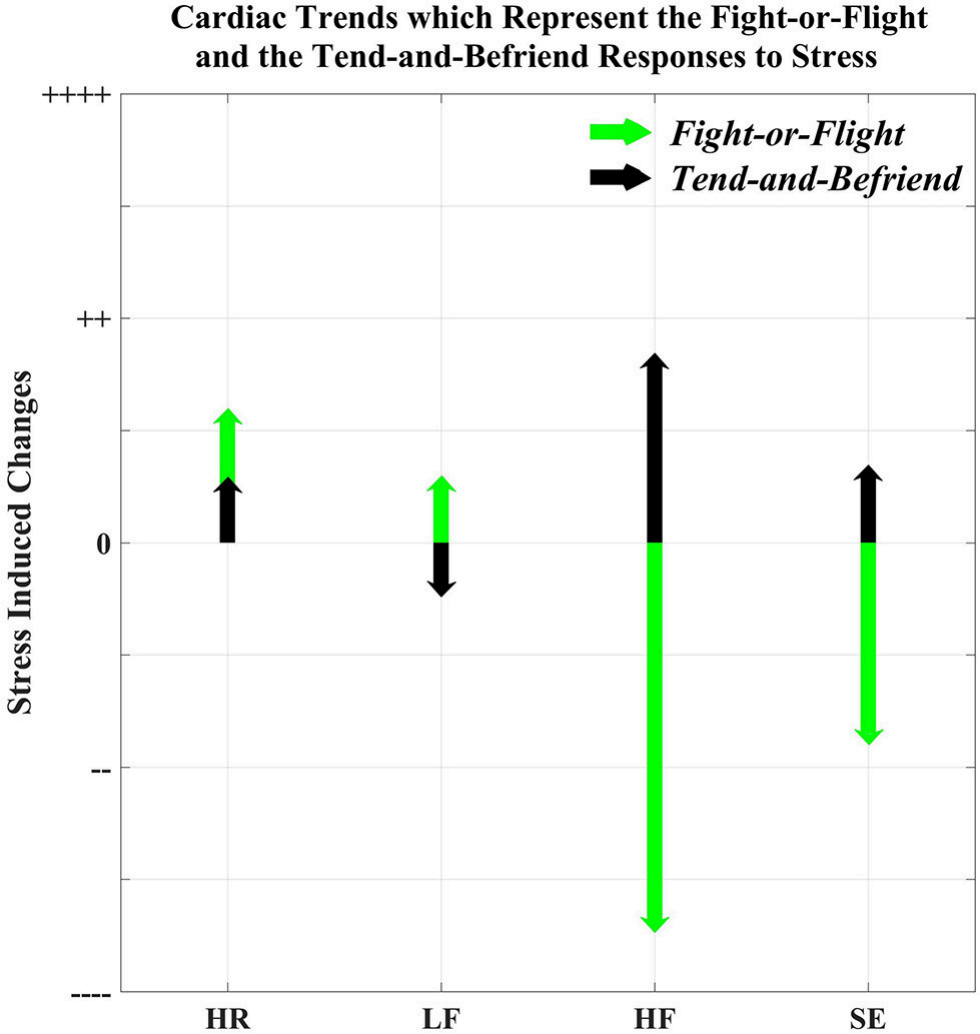
Cardiac trends representing the *fight-or-flight* and the *tend-and-befriend* responses, derived using the results reported in this study.

A female-specific stress response has been suggested by Taylor et al. (2000), who hypothesized that whilst males experience the conventional *fight-or-flight* response to stressors, females exhibit a *tend-and-befriend* response in which they employ social coping methods to combat stress. The tend-and-befriend response is driven by the action of estrogen and oxytocin (Taylor et al., 2000). Given the effects of estrogen on cardiac dynamics, the results from this study reveal, for the first time, cardiac trends which are likely to be specific to the *tend-and-befriend* response. It can be inferred that the *fight-or-flight* response is characterized by a large increase in HR, an increase in LF, a decrease in HF, and a decrease in HRV entropy; on the other hand, the *tend-and-befriend* response is characterized by a smaller increase in HR, a decrease in LF, an increase in HF, and an increase in HRV entropy, as illustrated in Figure 2.

Future studies must also incorporate endocrinological responses from the subjects, as the use of the calendar method to determine the menstrual phases of the female subjects, while appropriate for this pilot study, does not enable the validation of estrogen levels. This, in addition to the expansion of the female cohort to include females in the luteal phase, would enable a future comprehensive investigation into the female stress response.

## 5. Conclusion

We have investigated long-overlooked sex differences in the cardiac response to stress through temporal, spectral and nonlinear signatures of mental stress in heart rate variability (HRV) signals, recorded from 10 females in the follicular phase of the menstrual cycle, and 10 males. The cardiac responses were acquired during 15 min of rest and 15 min of a mental maths test, and the cardiac metrics include heart rate, normalized powers of the low frequency component, the normalized powers of the high frequency component, and the sample entropies of the HRV signals, accompanied by statistical comparisons. For rigor, we have also employed a new normalization procedure for the spectral components of HRV.

Every metric analyzed has revealed statistically significant differences (*p*-value < < 0.01) between the cardiac responses in the male and female subjects. In the males only, mental stress has been found to induce large increases in heart rate, increases in the power of the low frequency component of HRV, decreases in the power of the high frequency component of HRV, and decreases in the entropy of HRV. These trends have not been found in the females, suggesting that estrogen modulates the cardiac response to stress in females. Not only do the *results presented here radically challenge the practice of producing scientific hypotheses which have not accounted for sex*, but the stress-induced *increases* in the sample entropy of heart rate variability have never before been reported, thus challenging the common assumption that sample entropy is a reliable measure of signal complexity.

The results from this pilot study have established that the stress-induced cardiac trends which are commonly reported (increases in heart rate and the low frequency component of HRV) are the cardiac manifestations of the *fight-or-flight* response in males, whereas small increases in heart rate and large increases in the high frequency component of HRV may represent the female *tend-and-befriend* response. Following this proof-of-concept, further studies must employ females in the luteal phase of their menstrual cycle, and the collection of endocrinological parameters.

## Author contributions

The physiological data were recorded by TA and JX, the data analysis was completed by TA, under the supervision of DM, and the paper was written by TA and DM.

### Conflict of interest statement

The authors declare that the research was conducted in the absence of any commercial or financial relationships that could be construed as a potential conflict of interest.
